# Editorial: Current advances in precision microscopy

**DOI:** 10.3389/fmed.2025.1561485

**Published:** 2025-02-18

**Authors:** Rupert C. Ecker, Felicitas Mungenast, Quan Nguyen, Jyotsna Batra

**Affiliations:** ^1^Translational Research Institute, Queensland University of Technology, Brisbane, QLD, Australia; ^2^School of Biomedical Sciences, Faculty of Health, Queensland University of Technology, Brisbane, QLD, Australia; ^3^ARC Training Centre for Cell and Tissue Engineering Technologies (CTET), Queensland University of Technology, Brisbane, QLD, Australia; ^4^TissueGnostics GmbH, Vienna, Austria; ^5^Institute for Molecular Bioscience, The University of Queensland, Brisbane, QLD, Australia; ^6^Centre for Genomics and Personalised Health, Queensland University of Technology, Brisbane, QLD, Australia

**Keywords:** precision microscopy, image cytometry, organoid, single cell analysis, multiplexing, intravital microscopy, deep learning, machine learning

The field of precision microscopy has undergone a transformative evolution, driven by the integration of advanced imaging techniques, multiplex staining methods, as well as the application of computer vision techniques in artificial intelligence (AI) for image analysis. These innovations are not only enhancing our ability to visualize and analyze biological samples with unprecedented precision but are also paving the way for significant advancements in personalized medicine and precision diagnostics. The articles in this Research Topic collectively highlight the cutting-edge developments in precision microscopy, showcasing how these technologies are being leveraged to gain deeper insights into cellular and molecular processes, improve diagnostic accuracy, and ultimately, contribute to better clinical outcomes.

From the development of high-throughput imaging platforms that integrate machine learning algorithms for the analysis of 3D organoids and immune cell co-cultures, to the creation of novel software tools like Trapalyzer for the quantitative analysis of neutrophil extracellular trap formation, the advancements in precision microscopy are revolutionizing the way we study and understand complex biological systems across millions of cells, through 2D to 3D tissue spatial dimensions, incorporating temporal aspects. The integrative analyses of omics data and multiplex imaging, as discussed in one of the opinion articles, exemplifies the potential of these integrated approaches to provide a comprehensive understanding of cellular communication and its implications for disease progression and treatment.

The application of advanced light and fluorescence microscopy techniques, such as super-resolution microscopy and single-molecule imaging, is enabling researchers to visualize cellular processes with remarkable detail, aiding in the mechanistic understandings required for the development of targeted therapies. The integration of machine learning and deep learning techniques is further enhancing the accuracy and automation of biomarker identification and disease state analysis. Furthermore, multiplex staining methods are allowing for the simultaneous detection of multiple biomarkers, significantly improving the diagnostic capabilities at the molecular level.

The collective contributions of these articles underscore the transformative potential of precision microscopy in advancing our understanding of biological systems and improving clinical outcomes. By leveraging cutting-edge technologies and interdisciplinary approaches, researchers are making significant strides toward the realization of precision medicine. The integration of advanced imaging techniques, multiplex staining methods, and AI-driven analysis for patient samples, pre-clinical models and *in vitro* cell cultures is not only enhancing our ability to visualize and analyze biological structures with unprecedented precision but is also paving the way for significant advancements in personalized medicine and precision diagnostics.

As we continue to push the boundaries of what is possible with precision microscopy, it is essential to foster global collaboration, implement rigorous governance, and address ethical dilemmas head-on. By doing so, we can harness the full potential of these technologies for societal benefit, ensuring that precision microscopy aligns with humanity's highest aspirations. The future of precision microscopy is bright, and with continued innovation and interdisciplinary engagement, we are poised to make groundbreaking discoveries that will shape the future of medicine and healthcare.

1. Analysis of organoid and immune cell co-cultures by machine learning-empowered image cytometry.

This study presents a high-throughput imaging analysis platform that integrates automated imaging techniques and advanced image processing tools to analyze 3D organoids in co-cultures with immune cells (Stüve et al.). By employing machine learning algorithms, the platform can accurately identify and classify organoids, facilitating the study of organ development, immune disorders, and drug discovery.

2. Single cell analysis of the localization of the hematopoietic stem cells within the bone marrow architecture identifies niche-specific proliferation dynamics.

The publication combines flow cytometry with confocal microscopy and image analysis to investigate the location as well as the dynamics of HSCs (Mazzarini et al.). The results indicate that in aged mice, hematopoietic stem cells exhibit highly dynamic cycling and show a preference for interactions within the niche that directs their differentiation.

3. *In situ* Veritas: Combining omics and multiplex imaging to facilitate the detection and characterization of cell-cell interactions in tissues.

This opinion article discusses the potential of combining omics data with multiplex imaging to detect and characterize cell-cell interactions within tissues (Ritter). Such an approach can provide a comprehensive understanding of cellular communication and its implications for disease progression and treatment.

4. Human intravital microscopy in the study of sarcomas: an early trial of feasibility.

This brief report highlights the successful utilization of real-time intravital microscopy during the surgical removal of large sarcomas (Gabriel et al.). This method gives critical and immediate insight into data such as tumor vessel characteristics as well as their potential impact on administration and effectiveness of drug treatments.

5. Using quantitative single molecule localization microscopy to optimize multivalent HER2-targeting ligands.

This publication investigates the effect of different treatment regimens in cultured breast cancer cells on the organization of HER2 receptor (Wakefield et al.). The group used super-resolution microscopy in combination with other techniques to spatially analyse HER2 distribution. These findings give valuable new insights and may improve treatment strategies for HER+ breast cancer patients.

6. Trapalyzer: A computer program for quantitative analyses in fluorescent live-imaging studies of neutrophil extracellular trap formation.

Trapalyzer is a novel software tool designed for the quantitative analysis of neutrophil extracellular trap (NET) formation in live-imaging studies (Ciach et al.). This tool enhances the precision of NET quantification, providing valuable insights into the role of neutrophils in immune responses and inflammatory diseases.

7. A dual decoder U-net-based model for nuclei instance segmentation in hematoxylin and eosin-stained histological images.

The publication is introducing a dual decoder U-Net-based model for the segmentation of nuclei stained by hematoxylin in histological tissue images (Mahbod et al.). This algorithm is showing excellent segmentation performance and is the top-ranked method in the MoNuSAC post-challenge leaderboard.

8. Quantitative characterization of macrophage, lymphocyte and neutrophil subtypes within the foreign body granuloma of human mesh explants by 5 marker multiplex fluorescence microscopy.

This study emphasizes the potential for detailed characterization and spatial distribution analysis of immune phenotypes at inflammatory sites caused by surgical mesh (Klinge et al.). It offers insights into the complex interactions within this process and may contribute to the development of new treatment strategies.

9. Establishment and verification of neural network for rapid and accurate cytological examination of four types of cerebrospinal fluid cells.

This study introduces a deep neural network (DNN) for counting and classifying cerebrospinal fluid (CSF) cells, improving diagnostic efficiency (Jiang et al.). Using May-Grünwald-Giemsa stained images, the DNN accurately identifies key cell types and outperforms expert examination with 95% accuracy and an 86% reduction in turnaround time. The results demonstrate the potential of DNN classifier in clinical CSF cytology.

This Research Topic on *Current advances in precision microscopy* provides a snapshot of technological development in a highly innovative and vibrant scientific environment. We hope that this Research Topic will help spread the excitement the authors share with regards to this Research Topic and we wish our readers an insightful read!

